# Neuronal loss or dysfunction in patients with early Lyme neuroborreliosis: a proton magnetic resonance spectroscopy study of the brain

**DOI:** 10.1007/s00415-019-09359-0

**Published:** 2019-05-10

**Authors:** Adam Garkowski, Bożena Kubas, Marcin Hładuński, Joanna Zajkowska, Olga Zajkowska, Dorota Jurgilewicz, Radosław Zawadzki, Ewa Garkowska, Sławomir Pancewicz, Urszula Łebkowska

**Affiliations:** 1 Department of Radiology, Medical University of Białystok, M. Skłodowskiej-Curie 24A, 15-276 Białystok, Poland; 2 Independent Department, Laboratory of Molecular Imaging, Medical University of Białystok, Białystok, Poland; 3 Department of Infectious Diseases and Neuroinfections, Medical University of Białystok, Białystok, Poland; 4 Faculty of Applied Informatics and Mathematics, Warsaw University of Life Sciences SGGW, Warsaw, Poland

**Keywords:** Lyme neuroborreliosis, *Borrelia burgdorferi*, ^1^H-magnetic resonance spectroscopy,1H-MRS, Magnetic resonance spectroscopy

## Abstract

**Background:**

We hypothesized that since *Borrelia burgdorferi* causes systemic inflammation and infects the brain, it may lead to alterations in cerebral metabolism, as measured by ^1^H-magnetic resonance spectroscopy (^1^H-MRS). The purpose of our study was to determine whether ^1^H-MRS could detect brain metabolite alterations in patients with early Lyme neuroborreliosis (LNB) in normal-appearing brain tissue on the conventional magnetic resonance imaging (MRI).

**Methods:**

Twenty-six patients diagnosed with early LNB and twenty-six healthy volunteers as a control group have been involved in the study. All of them underwent routine MRI protocol using 3.0-T MRI scanner. ^1^H-MRS examinations were performed with repetition time (TR) = 2000 ms, and echo time (TE) = 135 ms. Single voxels were positioned in the anterior and posterior parts of the right and left frontal lobes.

**Results:**

We found a statistically significant decrease of the N-acetylaspartate/creatine ratio within the anterior part of the right and left frontal lobes (*p* ≤ 0.001 and *p* = 0.001 respectively) and in the posterior part of the right and left frontal lobes (*p* ≤ 0.001 and 0.031) in the patients with LNB.

**Conclusion:**

A significant reduction in NAA/Cr ratio in comparison with the controls suggests the presence of diffuse neuronal loss in patients with early LNB.

## Introduction

Lyme borreliosis (or Lyme disease) is a tick-transmitted multisystem inflammatory disease caused by the spirochete *Borrelia burgdorferi* sensu lato genospecies complex, and is the most common arthropod-borne disease in temperate regions of the northern hemisphere. In Europe, this disease affects approximately 65,500 patients annually. Lyme neuroborreliosis (LNB) is a disease of the nervous system, occurring in 10–15% of all Lyme borreliosis cases, and can occur at any stage of the disease, and may affect both the central (CNS) and peripheral nervous system. LNB is more common in Europe than in the United States, and typically manifests as Bannwarth’s syndrome including lymphocytic meningitis, cranial neuritis, and radiculoneuritis. These manifestations can occur separately or together [1, 2].The direct symptoms of CNS involvement vary widely, and may result, e.g., in symptoms such as a headache, difficulty with concentration, mood swings, disturbance of consciousness or Parkinson-like symptoms, and cerebrovascular complications like stroke caused by cerebral vasculitis [2, 3].

It is known that *B. burgdorferi* has a tropism for the meninges in the CNS and for connective tissues elsewhere in the body. Autopsy brain studies on patients with LNB are limited to single case reports or small case series. These studies showed, inter alia, diffuse demyelination of the cerebral and cerebellar white matter with diffuse astrocytosis [4], and rhombencephalopathy with microgliosis and obliterative inflammatory vasculitis associated with ischemic strokes [5]. In a recent study of an animal model of systemic inflammation, Ramesh et al., conducted an investigation to examine the role of inflammation on the CNS of *Rhesus macaques* infected intrathecally with *B. burgdorferi*. Histological studies of brain tissue from *R. Macaques* at necropsy performed early after infection, revealed, inter alia, leptomeningitis in the brain and spinal cord, vasculitis in the brainstem, focal inflammation in the CNS, and inflammation with neurodegeneration in the dorsal root ganglia that was concomitant with significant neuronal and glial cell apoptosis [6]. Neuroimaging studies are relatively insensitive in detecting the primary changes of *B. burgdorferi*-associated encephalitis. On conventional magnetic resonance imaging (MRI), the positive neuroimaging findings of patients with LNB are comparatively unusual. These findings are usually focal hyperintense lesions on T2-weighted images in the white matter of the brain or the nerve root or meningeal enhancement [7]. Sometimes imaging features may mimic primary demyelinating disease [8].

We hypothesized that since *B. burgdorferi* causes systemic inflammation and infects the brain, leading to impaired CNS function, it may lead to alterations in cerebral metabolism, as measured by in vivo ^1^H-magnetic resonance spectroscopy (^1^H-MRS).

^1^H-MRS is a non-invasive feasible method for in vivo quantification of several brain metabolites including N-acetylaspartate (NAA), choline-containing compounds (Cho), creatine (Cr), myo-inositol (mI) and glutamate–glutamine. Until now, ^1^H-MRS has been used as a research and clinical tool for detecting pathological changes visible or not yet visible on conventional MRI. The advantage of this method is the possibility to provide information about metabolite alterations in the brain, while MRI fails to reveal any morphological abnormalities [9].

The purpose of our study was to determine whether ^1^H-MRS could detect brain metabolite alterations in patients with early LNB in normal-appearing brain tissue on the conventional MRI study compared with healthy controls. We decided to use the long echo time (TE) of 135 ms for a more precise evaluation of changes in the NAA/Cr ratio.

## Methods

### Case definitions for LNB

According to the current European Federation of Neurological Societies (EFNS) guidelines for establishing a "definite" diagnosis of LNB, three conditions should be fulfilled: neurological symptoms suggestive of LNB without other obvious reasons, which occur in less than 6 months after the initial infection; cerebrospinal fluid (CSF) pleocytosis, and intrathecal synthesis of *B. burgdorferi* antibodies. If only two criteria are fulfilled, LNB is "possible" [10].

### Patients and control group

The study consisted of twenty-six patients with "definite" LNB according to the EFNS guidelines, hospitalized in the Department of Infectious Diseases and Neuroinfection of the Medical University of Białystok between July 2015 and December 2017. We did not include patients with "possible" LNB, because the diagnosis of LNB is speculative in this group. We also excluded patients suffering from LNB with any focal lesions in the brain on structural MRI (e.g., with T2-hyperintense foci). All our patients had early LNB (symptoms duration < 6 months). The patients’ age ranged from 19 to 65 years (sixteen males, ten females), with a mean age of 43 ± 14.3 years. The control group consisted of twenty-six healthy subjects (aged between 24 and 62 years, mean age 39.2 ± 10.8 years, eleven males, fifteen females) with no previous history of neurological dysfunction, and medical conditions affecting the brain, and with normal findings on MRI. Healthy volunteers were not taking any medication at the time of testing.

All patients with symptoms suggestive of LNB were tested for IgM and IgG antibodies to *B. burgdorferi* in serum and CSF by enzyme-linked immunosorbent assay (ELISA). In all patients, positive results obtained by ELISA were verified with confirmatory tests: Western blot/immunoblot (Borrelia IgM and IgG). Immunoblot for intrathecal production of specific antibodies against highly specific *B. burgdorferi* antigens was carried out for all patients (Virotech, Rüsselsheim, Germany). The simultaneous analysis of antibodies (IgG and IgM) against several *B. burgdorferi* antigens in serum and CSF was performed. Specific bands were compared and in the case where any band was expressed more in the CSF than in the serum, intrathecal synthesis was confirmed.

### Magnetic resonance imaging and ^1^H-MR spectroscopy acquisitions

**Fig. 1:**
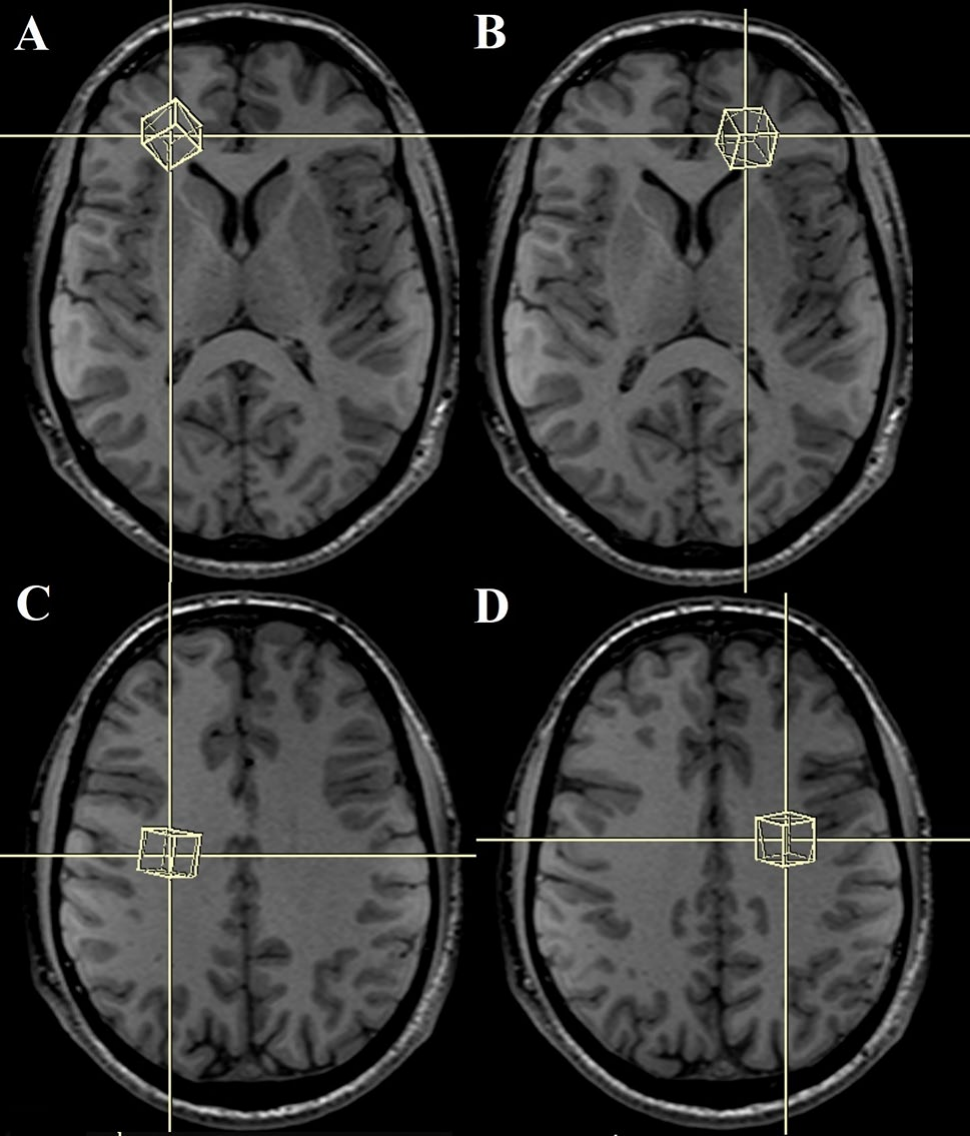
Sptatial localization of the VOI in the in the anterior (**a**, **b**) and posterior (**c**, **d**) parts of the right and left frontal lobes

MRI and ^1^H-MRS examinations were performed in a single session on a 3.0-T System (Biograph mMR, Siemens, Germany) using a 16-channel head matrix coil. The examinations were performed in the Independent Department, Laboratory of Molecular Imaging of the Medical University of Białystok. All patients with LNB underwent a routine brain MRI protocol to rule out any lesion or structural abnormality. The protocol included T1-weighted images (TR = 18 ms, TE = 4.92 ms, FoV = 230 mm, Base Resolution = 256, Slice thickness = 1 mm), T2-weighted images (TR = 3500 ms, TE = 117 ms, FoV = 200 mm, Base Resolution = 512, Slice thickness = 4 mm), fluid-attenuated inversion recovery (FLAIR) (TR = 2500 ms, TE = 94 ms, TI = 2500 ms, FoV = 240 mm, Base Resolution = 256, Slice thickness = 4 mm), diffusion-weighted images (DWI) (TR = 8700 ms, TE = 82, FoV = 240 mm, Base Resolution = 132, Slice thickness = 4 mm, *b* value 1 = 0 s/mm^2^, *b* value 2 = 500 s/mm^2^, *b* value 3 = 1000 s/mm^2^, *b* value 4 = 1500 s/mm^2^), and T1-weighted images with contrast medium at the end. ^1^H-MRS was obtained before T1-weighted images with contrast medium administration. Healthy volunteers underwent the same brain MRI protocol, but without T1-weighted images with contrast medium administration. The median duration from onset of symptoms until diagnosis and initiation of treatment was 15 days. The patients received antibiotic treatment regimens consisting of ceftriaxone. All patients underwent MRI and ^1^H-MRS examinations shortly before or at the beginning of antibiotic therapy. Localized ^1^H-MRS examinations were performed with a point-resolved spectroscopy (PRESS) sequence. The spectral acquisition parameters were TE of 135 ms, repetition time (TR) of 2000 ms, averages of 128, and 1200 Hz bandwidth. The scan time was 4 min 24 s. The volume of interest (VOI) was located in four regions of the brain: bilaterally in the anterior and posterior parts of the right and left frontal lobes (VOI size: 15 × 15 × 15 mm). We chose frontal lobes for three main reasons: (1) patients with LNB often suffer from difficulty with concentration, mood swings and inability of spontaneous thinking; the dysfunction of frontal lobes of the brain can lead to such changes (2) apart from that, we placed four voxels (VOI) frontally, because in these areas, T2-hyperintense foci tend to occur more often, than in other parts of the brain in patients with LNB (3) additionally, one positron emission tomography (PET) study reported diffuse hypometabolism that included, e.g., frontal lobes. Importantly, in the aforementioned study, there were no metabolic changes in cerebellum [11]. Another single-photon emission computed tomography (SPECT) study demonstrated decreased perfusion in the frontal lobes in 13 patients with LNB [12]. The positioning of VOI is shown in Fig. 1. The localization of VOI was confirmed by three orthogonal MR images in axial, sagittal, and coronal planes. The voxels were localized manually by a trained and experienced neuroradiology technician under my control using T1-weighted sections: in axial, coronal and sagittal planes, reducing the inclusion of CSF. The first two voxels were located on the opposite sides of the cerebral hemispheres symmetrically in anterior part of the frontal lobes (forward and laterally in relation to the anterior horns of the lateral ventricles), and encompassed mainly the frontal white matter. The second two voxels were placed on the opposite sides of the cerebral hemispheres symmetrically exclusively in centrum semiovale of the frontal lobes (above and laterally in relation to the body of the lateral ventricles). The locations of each voxel were chosen carefully to ensure that each voxel contained mainly white matter and in the same areas. No structural pathologies in the areas of VOI on performed structural MRI were observed. Data were acquired and areas of the three peaks of NAA at 2.02 ppm, Cr at 3.02 ppm, and Cho at 3.22 ppm were measured. The metabolite ratios of NAA/Cr, and Cho/Cr were calculated for each VOI, ^1^H-MRS spectra were analyzed using the Linear Combination of Model spectra (LCModel) (version 6.3), using the unsuppressed water signal as a concentration reference [13]. Only metabolites with standard deviations of less than 15% were included in the analysis (Cramer-Rao lower bounds, as determined by LCModel), this being a reliable indicator of good-quality spectra.

### Statistical analysis

The analysis was conducted with Stata 15 software. The significance of metabolite ratio differences between patients with LNB and control group were estimated with the unpaired *t* test. Since the pool of patients with LNB and control group is limited we bootstrapped the *p* values for the two tailed *t* test with 10,000 replicates. A value of *p* < 0.05 was considered as statistically significant. Differences between patients with LNB and healthy controls were tested separately for each VOI and each metabolite. We confirmed the robustness of the results with the parametric (one-way ANOVA) and nonparametric methods (Kruskal–Wallis test, Dunn’s pairwise comparison test, Wilcoxon rank-sum test).

## Results

### Demographics and clinical data

Patient characteristics are shown in Table 1. Cranial nerve palsy and meningitis were the dominant clinical symptoms of LNB. Three patients demonstrated contrast enhancement of cranial nerves, which corresponded to clinical findings. The remaining patients had normal structural MRI.

**Table 1:** Epidemiological and clinical characteristics among 26 patients with Lyme neuroborreliosis

Characteristic	No. of patients (%)
Mean age ± SD, median age, years (range)	43 ± 14.3, 44 years (19–65)
Male, sex	16 (62%)
History of tick bite	8 (32%)
History of erythema migrans	4 (16%)
Median duration of symptoms before lumbar puncture (< 6 months), weeks (range)	2 (0.4–18)
Clinical features of LNB	
Cranial nerve palsy^a^	14 (56%)
Facial nerve	14
Abducens nerve	1
Meningitis	13 (50%)
Meningoradiculoneuritis	6 (24%)
Other	3 (12%)
Cognitive and behavioral data	
Anxiety	9 (34.6%)
Memory impairment	5 (19.2%)
Concentration difficulties	5 (19.2%)
Fatigue	7 (27%)
CSF analysis	
Median cell count (range)	110.5 cells/μL (15–333)
Median protein level (range)	740 mg/L (432–2660)
Median glucose level (range)	2.89 mmol/L (1.61–3.61)

^a^ Includes 12 patients with unilateral peripheral facial palsy and 2 patients with bilateral peripheral facial palsy. Among 14 patients with facial nerve palsy, 2 patients had central facial palsy

### ^1^H-MRS

**Fig. 2:**
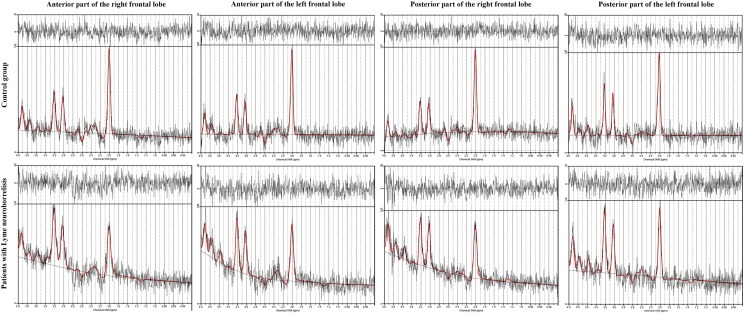
Sample spectra for healthy controls (upper panel) and patients with LNB (lower panel) from the four volumes of interest

MR spectra were successfully acquired in all patients and healthy controls. There was no correlation between NAA/Cr and Cho/Cr ratios and age in patients or controls. Table 2 summarized the mean and standard deviation of the metabolite ratios in the six regions of the brain in twenty-six patients diagnosed with LNB and 26 healthy control subjects. NAA/Cr ratio was significantly lower in the anterior part of the right and left frontal lobes (*p* ≤ 0.001 and *p* = 0.001 respectively), and in the posterior part of the right and left frontal lobes (*p* ≤ 0.001 and 0.031 respectively). There was no statistically significant change of Cho/Cr ratio within all regions. We found differences between the MR spectra obtained in patients diagnosed with early LNB and normal conventional MRI of the healthy control subjects. Representative examples of normal and abnormal spectra are shown in Fig. 2. There is a relative reduction of the NAA peak when compared with the Cr peak.

**Table 2:** Metabolite ratios for patients with LNB and control group

Location of the voxel	NAA/Cr			Cho/Cr		
	Patients	Control group	*p* value^a^	Patients	Control group	*p* value^a^
Anterior part of the right frontal lobe	1.777 ± 0.319*	2.079 ± 0.249	≤ 0.001	0.371 ± 0.061	0.375 ± 0.077	0.868
Anterior part of the left frontal lobe	1.832 ± 0.270*	2.096 ± 0.300	0.001	0.386 ± 0.044	0.393 ± 0.048	0.584
Posterior part of the right frontal lobe	2.254 ± 0.286*	2.639 ± 0.256	≤ 0.001	0.379 ± 0.048	0.377 ± 0.050	0.855
Posterior part of the left frontal lobe	2.259 ± 0.346*	2.449 ± 0.268	0.031	0.376 ± 0.058	0.362 ± 0.038	0.307

Values are given as means ± SD

^*^p < 0.05

^a^*p* value for comparison between patients with LNB and control group

## Discussion

Nervous system involvement is a common feature of *B. burgdorferi* infection. Early after systemic infection, *B. burgdorferi* invades CNS along both the blood vessels and other structures such as the peripheral nerves. The symptoms of LNB are triggered by a focal or diffuse neuronal dysfunction, which may be caused by several mechanisms [14]. Infection with *B. burgdorferi* could directly or indirectly cause systemic inflammations by inducing an immunological response to the disease and metabolic imbalance. On the one hand, *B. burgdorferi* may directly wield neurotoxicity by adhering to neural and glial cells. On the other hand, indirect effects of *B. burgdorferi* on neurons involve the activation of glial cells, resulting in the production and release of neurotoxic cytokines such as TNF-α and IL-6, which may cause neuronal damage or dysfunction [14–17].

In patients with early LNB, conventional MRI is usually normal. According to one study, about 17% of patients with LNB had abnormal findings on MRI. The most common MRI findings are multiple and small subcortical and periventricular white matter lesions hyperintense on T2/FLAIR. Nerve root or meningeal enhancement is less common [7]. To the best of our knowledge, our study represents the largest series of patients with LNB investigated with ^1^H-MRS. The results demonstrated a statistically significant decrease of the NAA/Cr ratio within the anterior part of the right and left frontal lobes, and in the posterior part of the right and left frontal lobes. The reduction was detected in the early phase of illness. However, no significant difference was noted for Cho/Cr ratio in the two groups using localized ^1^H-MRS at 3.0 T. Therefore, the Cho/Cr ratio may not be as sensitive as the NAA/Cr ratio in detecting early changes in the brain of patients with LNB. In our study, significantly decreased NAA/Cr ratio was found in the areas of the brain which were normal on conventional MRI. Accordingly, we conclude that the reduction of NAA/Cr ratio may reflect neuronal dysfunction or early neuronal damage without visible structural abnormalities of the brain, which may signify latent encephalopathic changes undetectable by MRI in patients with early LNB. Since NAA is synthesized in neuronal mitochondria and found exclusively in the nervous system, it has been proposed as a marker of neuronal density and mitochondrial activity. The reduction of NAA has been reported in certain disorders with neuronal loss or dysfunction, including brain metabolic disorders, neurodegenerative diseases, ischemia, demyelinating diseases, systemic lupus erythematosus, rheumatoid arthritis and some infections, such as herpes simplex encephalitis, HIV and HCV infection [9, 18–24]. There is only one study describing the spectroscopic changes in the brain in patients with LNB. In that study, VOI was placed in the single region of the brain (frontal lobe) in twelve patients with various stages of LNB (early and late). The authors demonstrated significant increase in Cho/Cr and Lip/Cr ratios compared with controls (*p* < 0.001). In contrast to our study, no statistically significant abnormality was found in mean NAA/Cr ratio. In the aforementioned study, ^1^H-MRS spectra were acquired at the shortest TE (= 35 ms) [25]. The main aim of our study was to quantify alterations in neuronal activity and/or damage, which is mainly reflected by the signal of NAA. In ^1^H-MRS, there are two basic techniques: (1) the “short-TE” approach (e.g., TE = 35 ms) with more visible metabolites (myo-inositol, glutamate and glutamine), the disadvantage of a distorted baseline (due to macromolecule signal contribution) and worse quantification of NAA: (2) the “long-TE” approach (TE = 135 ms) with more accurate quantification of the NAA, and Cho metabolites. With smaller baseline distortions [26]. For a more precise evaluation of changes in the NAA/Cr ratio, we decided to choose the long-TE approach. In contrast to our study, the authors of the aforementioned study reported a significant reduction in Cho/Cr ratio in the frontal lobes [25]. The relevance of this discrepancy is uncertain. One possible explanation is the difference of technical factors such as location and size of VOI and different TE (35 ms vs 135 ms). In addition, our study was performed using a scanner with higher magnetic field strength (3.0 T) compared with 1.5-T MRI scanner in the mentioned study, which may explain the differences. Another possible explanation is that a decrease in NAA/Cr ratio without concominant increase in Cho/Cr ratio may reflect either neuronal dysfunction or an initial loss without a (yet) significant neuronal damage reflected by an increase in Cho/Cr ratio at an early stage of LNB.

Our study has some limitations. First, this study involved a relatively small number of patients at one institution; thus, it is necessary to accumulate more patients in future research. Second, ^1^H-MRS data were only obtained from the few regions of the brain due to time constraints, because patients could not tolerate the long acquisition time required to obtain additional ^1^H-MRS data from other sides (e.g., from cerebral cortex or occipital lobes).

## Conclusion

In conclusion, localized ^1^H-MRS at 3.0 T in multiple regions demonstrates a significant reduction in NAA/Cr ratio in patients with early LNB in comparison with the healthy controls, indicating the presence of neurological damage or dysfunction likely caused by neuronal injury due to *B. burgdorferi* infection. ^1^H-MRS is more sensitive than conventional MRI for evaluation of CNS involvement in early LNB.
